# Endocrinologic Dysfunctions and Neuropsychiatric Sequelae in Pediatric Patients With a History of Central Nervous System Infection (ENDLESS): A Prospective Monocentric Study

**DOI:** 10.1097/INF.0000000000004645

**Published:** 2024-12-24

**Authors:** Giorgio Sodero, Clelia Cipolla, Anna Camporesi, Laura Martino, Simonetta Costa, Zemira Cannioto, Paolo Frassanito, Gianpiero Tamburrini, Chiara Veredice, Luca Maggio, Daniel Munblit, Francesca Raffaelli, Marco Piastra, Giuseppe Zampino, Piero Valentini, Danilo Buonsenso

**Affiliations:** From the * Department of Woman and Child Health and Public Health, Fondazione Policlinico Universitario A. Gemelli IRCCS, Rome, Italy; † Pediatric Anesthesia and Intensive Care, V. Buzzi Children’s Hospital, Milano, Italy; ‡ Division of Neonatology, Department of Pediatrics, University Hospital A Gemelli, Catholic University of the Sacred Heart, Rome, Italy; § Neonatology Unit, “San Camillo-Forlanini” Hospital, Rome, Italy; ¶ Pediatric Neurosurgery, Fondazione Policlinico Universitario A. Gemelli IRCCS, Rome, Italy; ∥ Division of Care in Long Term Conditions, Florence Nightingale Faculty of Nursing, Midwifery and Palliative Care, King’s College London, London, UK; ** Department of Paediatrics and Paediatric Infectious Diseases, Institute of Child’s Health, Sechenov First Moscow State Medical University (Sechenov University), Moscow, Russia; †† Research and Clinical Center for Neuropsychiatry, Moscow, Russia; ‡‡ UOC Malattie Infettive, Dipartimento di Scienze Mediche e Chirurgiche, Fondazione Policlinico Universitario Agostino Gemelli IRCCS, Rome, Italy; §§ Pediatric ICU and Trauma Center, Fondazione Policlinico Universitario “A. Gemelli” IRCCS, Rome, Italy; ¶¶ Institute of Anesthesia and Intensive Care, Catholic University of the Sacred Heart, Rome, Italy; ∥∥ Centro di Salute Globale, Università Cattolica del Sacro Cuore, Roma, Italia; *** Area Pediatrica, Dipartimento di Scienze della Vita e Sanità Pubblica, Università Cattolica del Sacro Cuore, Roma, Italia.

**Keywords:** central nervous system infections, pediatric endocrinology, pediatric infectious diseases, meningitis, encephalitis

## Abstract

**Introduction:**

Central nervous system (CNS) infections represent some of the most critical pediatric health challenges, characterized by high mortality rates and a notable risk of long-term complications. Despite their significance, standardized guidelines for endocrinological follow-up of CNS infection survivors are lacking, leading to reliance on the expertise of individual centers and clinicians.

**Materials and Methods:**

Prospective monocentric observational study conducted at the Fondazione Policlinico Universitario Agostino Gemelli in Rome, Italy. It included patients with a history of CNS infection, admitted to various pediatric departments of the hospital. The participants were selected based on a coded diagnosis of CNS infection and had completed their follow-up at the Pediatric Endocrinology Day Hospital after October 2019.

**Results:**

Eighty participants were included, comprising 53 patients with a prior CNS infection and 27 healthy controls, with a median age of 7.4 years (range 3.6–12.3 years). Endocrinologic alterations were detected in 13 patients, with 8 cases in those who had meningitis, 4 in encephalitis survivors, and 1 in a patient with a cerebral abscess. Patients with a history of CNS infections were shorter compared with healthy controls (*P* = 0.027). Moreover, those who had meningitis exhibited an increased risk of developing epilepsy (*P* = 0.01), neurosensory disabilities (*P* = 0.034) and the need for ventriculoperitoneal shunt insertion (*P* = 0.006). Patients with bacterial CNS infections were more prone to neurosensory and endocrine dysfunctions compared to those with viral or other infections. Significant differences were observed in hormone levels between previously infected patients and controls, specifically in TSH (*P* < 0.001), ACTH (*P* = <0.001), and cortisol (*P* = 0.019). IGF-1 levels were considerably lower in the infection group, both in absolute terms and when adjusted for sex and age (*P* < 0.001). The regression analysis suggested that the reduction in IGF-1 was more pronounced the earlier the CNS infection occurred, irrespective of infection type.

**Conclusions:**

Our study found several endocrinologic imbalances in children who survived CNS infections.

Central nervous system (CNS) infections are among the most severe pathologies during pediatric age, presenting a high mortality rate and a significant incidence of long-term sequelae.^1–3^

The precise incidence of endocrinologic sequelae in patients with meningoencephalitis contracted in pediatric age is not known^4^; studies conducted on adult populations have shown hormonal deficiencies may remain occult in the acute phase of infection, manifesting in the long-term follow-up, even years after recovery from the infectious episode.^5,6^ It is reported in the literature that the most severe forms with a suggestive symptomatic clinical manifestation at diagnosis are more frequently associated with long-term complications.^1^

In pediatric patients, CNS infections can lead to potentially life-threatening endocrine sequelae, such as adrenal insufficiency,^5^ and may negatively affect the production of other pituitary tropic hormones, causing developmental alterations (growth hormone deficiency), delayed puberty due to reduced gonadotropin production, or central hypothyroidism.^4,5^ Nonetheless, studies conducted on pediatric patient populations with a history of CNS infection have shown that the onset of endocrinopathies is rare following pediatric CNS infections, likely due to the greater intrinsic resilience of the pituitary gland compared to adult populations.^4^ The precise incidence of these sequelae is not known, as the data in the literature is primarily derived from patients with symptomatic endocrinological deficits, while subclinical forms may remain latent for many months or years.^7^

Regarding the association between hormonal deficiencies and the underlying infectious agent, limited information is available for certain pathogens, such as *Mycobacterium tuberculosis*, which can cause hypothalamic-pituitary dysfunction in up to 20% of cases.^8^

Furthermore, there is currently a recommendation for the endocrinologic follow-up of patients with CNS infection; therefore, management relies on the experience of individual centers and clinicians.^9^

Based on current gaps in the literature, we proposed this study to analyze the incidence of endocrinological dysfunctions in children with a history of infectious involvement of the CNS.

## MATERIALS AND METHODS

This is a prospective, observational, single-center study conducted on patients with a previous infection of the CNS, hospitalized in all the Pediatric Departments of the *Fondazione Policlinico Universitario Agostino Gemelli* located in Rome, Lazio, Italy.

We recruited all children previously hospitalized for a CNS infection, selected based on the inclusion and exclusion criteria outlined below.

Patients were selected from children hospitalized with a coded diagnosis of CNS infection and with follow-up at the Pediatric Endocrinology Day Hospital, starting in October 2019.

In our center, multidisciplinary pediatric, endocrinological and infectious disease follow-up is part of the routine management of these patients; all children with a history of CNS involvement undergo thorough medical history and baseline hormonal assays to early identify signs and symptoms of acquired hypopituitarism: patients with abnormal baseline laboratory findings undergo dynamic stimulation tests^9^ to confirm or exclude suspected hormonal deficiency; children with clinical signs suggestive of endocrinopathy are directly subjected to second-level tests to assess hormone secretion. In all other cases, patients continue periodic follow-up, annually for a period of at least 3 years, at the Pediatric Endocrinology Day Hospital, to assess the possible late onset of hormonal complications.

### Ethics Committee

The study was reviewed and approved by the Human Research Ethics Committee of the Fondazione Policlinico Universitario A. Gemelli IRCCS of Rome, Italy (ID 6307). The study was conducted in accordance with the Declaration of Helsinki and its subsequent amendments. No personal or identifiable data were collected during the conduct of this study. All parents of the children signed informed consent; they could choose not to participate in the study or to withdraw their consent at any time.

### Inclusion Criteria

Children admitted in all the Pediatric Departments of the *Fondazione Policlinico Universitario Agostino Gemelli* from October 2019 and the following inclusion criteria:

1. Age >30 days at the time of endocrinological follow-up.
2. Previous coded diagnosis of CNS infection (meningitis, encephalitis or brain abscess), and defined by the presence of signs and symptoms compatible with neurological involvement and positive microbiological tests (cerebrospinal fluid culture, cerebrospinal fluid chemical and physical examination, positive film array on cerebrospinal fluid, brain tissue culture if applicable) or, in case of cerebral abscesses, positive brain computed tomography and/or magnetic resonance imaging (MRI).
3. No clinical/laboratory evidence of previous endocrine disorders in the period preceding the CNS infection.
4. Complete postinfectious follow-up (infectious evaluation, endocrinological evaluation and follow-up blood tests).

As a control group, we included otherwise healthy children younger than 18 years of age who never had CNS infections and were seen in the pediatric endocrinological unit of our hospital for a suspected short stature that underwent endocrinological testing that was normal.

### Exclusion Criteria

1. Age >18 years.
2. Evidence of congenital CNS alterations diagnosed on radiologic examinations (brain CT, brain MRI).
3. Complex genetic syndromes.
4. Patients with ventriculoperitoneal or ventriculoatrial shunt, except for patients who had the device placed following the infectious episode of the CNS.

### Outcomes

The main objective of the study was to evaluate the incidence of endocrinological alterations in a cohort of patients with a previous diagnosis of CNS infection.

The secondary objectives of the study were:

1. To evaluate the incidence of various endocrinologic alterations by comparing the group of patients with meningitis with those with encephalitis and with patients with brain abscesses.
2. To conduct, if possible, a subgroup analysis by comparing the incidence based on the individual identified etiological agent.
3. To evaluate the incidence of various endocrinological alterations by comparing the group of patients with CNS infections diagnosed in neonatal age (less than 28 days), infancy (29 days to 2 years), or at 3 years of age or older.

### Methodology

After confirming the inclusion and exclusion criteria, the parents of the patients were informed about the objectives of our study and provided written informed consent. The children underwent the standard multidisciplinary follow-up for CNS infection. Subsequently, the following information was retrieved from the patients’ medical records:

1. General physiologic and pathologic history, including predisposing factors for CNS infections and/or hormonal alterations.
2. Laboratory tests, including hormone levels before CNS infection, if available.
3. Microbiological tests conducted during hospitalization for CNS infection (cerebrospinal fluid culture, chemical and physical examination of cerebrospinal fluid, positive film array on cerebrospinal fluid, brain tissue culture if applicable).
4. Follow-up at the Pediatric Endocrinology Day Hospital within 3 years following hospitalization for acute CNS infectious episode, including: (1) Pediatric examination with a complete recording of auxological parameters, vital signs, and blood pressure. (2) Pediatric infectious disease visit (aimed at identifying possible sequelae of CNS infection through medical history). (3) Pediatric endocrinology visit (aimed at identifying possible hormonal alterations resulting from CNS infection through medical history). (4) Laboratory tests (sodium, potassium, plasma osmolarity, glucose, insulin, TSH, fT3, fT4, TSH, anti-TPO and TG antibodies, cortisol, ACTH, renin, aldosterone, IGF-1, GH, FSH, LH, estradiol or testosterone, prolactin).

The measurement of renin, aldosterone, insulin and glucose, and the calculation of HOMA-IR levels were conducted as they are included in the standard hormonal assays provided by our center for patients with suspected or confirmed endocrinopathies, despite these values are not directly connected with pituitary function and secretion.

1. Corticotropin deficiency is diagnosed when morning cortisol concentrations are less than 100 nmol/L.
2. Central hypothyroidism is diagnosed when concentrations of free thyroxine are decreased and TSH levels are low to slightly elevated.
3. Hypogonadism in childhood typically presents no clinical symptoms until the onset of puberty, at which time it often manifests with delayed or absent puberty.
4. Arginine vasopressin deficiency is diagnosed based on alterations in plasma osmolarity and levels of sodium and potassium; definitive diagnosis requires a water deprivation test, which was performed only in the presence of a high clinical suspicion.
5. Hyperprolactinemia is diagnosed if basal levels of prolactin are elevated and a subsequent stimulation test (thyrotropin-releasing hormone test) yields pathological results with an abnormal prolactin response (exaggerated or blunted).

### Statistical Analysis

Descriptive data are shown as absolute numbers and percentages for categorical variables and median and interquartile range (IQR) for continuous ones. Associations between variables were tested with Pearson’s χ^2^ test in the case of categorical variables and with the Kruskal–Wallis test in the case of continuous variables. In this latter case, Dunn’s post hoc test was conducted with Bonferroni correction as needed. Regression models were then applied to the outcome variables of interest. Best models were chosen including the clinically relevant variables and according to their R-squared. Variables included in the final model were checked for multicollinearity by variance inflation factor evaluation, and the linearity in the logit of continuous variables was assessed by visual inspection of the logit plot. Possible final models were evaluated by comparing their respective akaike information criterion and Bayesian information criterion.

All statistical tests were two-sided, and the level of statistical significance was set at 0.05. Reported *P* values have not been corrected for multiple hypothesis testing. Data were analyzed with Stata v. 18 BE (Statacorp, LLC, TX).

## RESULTS

In our study, we included 80 patients (53 with previous CNS infections and 27 healthy controls) with a median age of 7.4 (3.6–12.3) years. The general demographic and anthropometric characteristics of our cohort are reported in Table 1.

**TABLE 1.** Demographic Data of our Cohort of Patients; Data are Presented as Median (IQR) for Continuous Measures, and n (%) for Categorical Measures

	Controls	CNS Infections	
	N = 27	N = 53	*P* value
Age at evaluation (years)	9.2 (5.3–11.8)	6.8 (3.0–13.3)	0.16
Female sex	9w (33.3%)	25 (47.2%)	0.24
Height SD	0.52 (−0.53 to 0.83)	−0.25 (−0.85 to 0.73)	**0.027**
Height <2 SD	0.0 (0%)	7 (13.2%)	**0.049**
Weight SD	0.6 (−0.1 to 1.3)	0.1 (−0.8 to 0.8)	0.054
BMI SD	0.58 (−0.40 to 1.46)	0.19 (−0.68 to 1.01)	0.19
Puberty (number)	12 (44.4%)	17 (32.1%)	0.28

Bold value indicates statistically significant values.

BMI indicates body mass index.

Patients with a previous CNS infection, despite presenting demographic characteristics like those of the controls, had a shorter height compared to healthy patients (*P =* 0.027), assessed using standard deviations for sex and age calculated based on reference percentiles.

Among the 53 children with prior infection, 21 (3.9.6%) had a history of bacterial meningitis, 22 (41.5%) had a history of encephalitis and 10 (189%) had a previous episode of cerebral abscess. Median age was 6.8 (3.0–13.3) years, with 16 children (30.2%) under 30 days of age.

All patients underwent lumbar puncture for cerebrospinal fluid examination. The causative agent of the infection was identified through cerebrospinal fluid culture or molecular analysis of cerebrospinal fluid.

The pathogen most frequently isolated, in 5 cases, was *Streptococcus intermedius* (11.32%); in all cases, it was responsible for brain abscess. Within meningitis cases, the bacterium most frequently isolated was *Streptococcus pneumoniae* (5 cases, 9.43%), followed by *Escherichia coli* (4 cases, 7.55%), while the virus most frequently isolated was Paraechovirus (4 cases, 7.55%). In 5 cases of encephalitis, it was not possible to identify the etiologic agent; based on the clinical characteristics and outcomes observed during follow-up, in all 5 cases, a viral pathogen underlying the condition was hypothesized. Two cases (3.77 %) were caused by *Mycobacterium tuberculosis*, while only 1 case (1.89%) was caused by a fungus (*Aspergillus niger*). More details on the isolated etiologic agents are reported in Tables and Figures, Supplemental Digital Content 1–29, http://links.lww.com/INF/F912.

All patients required hospitalization and intravenous therapy; at least 1 antibiotic, either as anti-infective therapy or as prophylaxis, was administered in 45 cases (84.9%), while an antiviral (acyclovir) was administered in 14 children (26.4%). Steroid therapy for managing CNS infection was prescribed in 35 patients. ACTH levels were not significantly different between patients treated with steroids and those not (treated with steroids: 16 (12–21), not treated with steroids: 19 (14–27), *P =* 0.14.

Median follow-up time was 1.21 (0.68–3.19) years; analyzing the multidisciplinary follow-up data of these patients, regarding disability following the acute infection episode, 1 patient with previous bacterial meningitis underwent tracheotomy, 2 children with previous meningitis required percutaneous endoscopic gastrostomy, and 10 (8 previous meningitis and 2 previous cerebral abscess) underwent ventriculoperitoneal shunt insertion.

### Type of CNS infection

Patients with previous meningitis had a significantly higher risk of developing epilepsy (*P =* 0.01), neurosensory disabilities (*P =* 0.034) and ventriculoperitoneal shunt insertion (*P =* 0.006). Comparing the various infection subtypes among themselves and with the control group, a significant correlation was found between the type of infection and neurological outcomes. More information is provided in Table 2.

**TABLE 2.** Comparison Between Auxological Parameters, Neurologic Outcomes and Endocrine Dysfunctions in our Cohort of Patients

	Meningitis	Encephalitis	Abscess	Controls	
	N = 21	N = 22	N = 10	N = 27	*P* value
Pubertal age	7 (33.3%)	3 (13.6%)	7 (70.0%)	12 (44.4%)	**0.014**
Age at first endocrinologic evaluation	6.7 (3.3–10.4)	4.1 (1.4–8.7)	14.4 (10.5–16.6)	9.2 (5.3–11.8)	**<0.001**
Height SD	−0.6 (−2.1 to −0.0)	0.0 (−0.3 to 0.8)	−0.3 (−0.6 to 1.0)	0.5 (−0.5 to 0.8)	**0.012**
Height <2 SD	6 (28.6%)	1 (4.5%)	0 (0.0%)	0 (0.0%)	**0.002**
Weight SD	−0.2 (−1.1 to 0.4)	0.1 (−0.6 to 0.9)	0.5 (−0.3 to 1.3)	0.6 (−0.1 to 1.3)	**0.044**
BMI SD	−0.2 (−0.9 to 1.0)	0.2 (−0.7 to 0.9)	0.6 (−0.4 to 1.6)	0.6 (−0.4 to 1.5)	0.33
Epilepsy	7 (33.3%)	1 (4.5%)	0 (0.0%)	0 (0.0%)	**<0.001**
Motor dysfunction	9 (42.9%)	5 (22.7%)	6 (60.0%)	0 (0.0%)	**<0.001**
Cognitive dysfunction	7 (33.3%)	2 (9.1%)	1 (10.0%)	0 (0.0%)	**0.006**
Neurosensorial dysfunction	7 (33.3%)	1 (4.5%)	1 (10.0%)	0 (0.0%)	**0.002**
Endocrine dysfunction	8 (38.1%)	4 (18.2%)	1 (10.0%)	0 (0.0%)	**0.005**
Central endocrine dysfunction	8 (38.1%)	3 (13.6%)	1 (10.0%)	0 (0.0%)	**0.003**
Symptomatic endocrine dysfunction	6 (28.6%)	1 (4.5%)	1 (10.0%)	0 (0.0%)	**0.008**
General neurologic deficit	13 (61.9%)	5 (22.7%)	6 (60.0%)	0 (0.0%)	**<0.001**

Bold value indicates statistically significant values.

BMI indicates body mass index.

Furthermore, comparing the type of infection and evaluating the incidence of endocrinological outcomes, we found that patients with bacterial infections have a higher risk of developing neurosensory and endocrine dysfunctions compared with patients with viral or other types of infections (*P =* 0.004 and *P =* 0.02); see Tables and Figures, Supplemental Digital Content 1–29, http://links.lww.com/INF/F912 for more information.

### Age at the time of infection

We subsequently subclassified the patients based on the age at which they contracted the CNS infection, resulting in 3 groups (neonates less than 28 days, infant toddlers between 29 days and 2 years and children >2 years); it is interesting to remark, newborns showed a higher rate of meningitis compared to children while abscesses were found only in children. Encephalitis was also more represented in the newborns and toddlers’ group than in the child age.

According to our classification criteria, no significant difference in the incidence of endocrine and/or neurologic dysfunctions was found among the 3 groups.

Endocrinologic alterations were diagnosed in 13 patients; of these, 8 were diagnosed in patients with previous meningitis, 4 in children with previous encephalitis and 1 in a patient with previous cerebral absces*s.*

Except for 1 case of thyroiditis, all other endocrinological alterations (12/13) had a central origin, possibly correlated with the previous infectious episode. Endocrine deficiency was symptomatic in 8 patients, while in the remaining 5 cases, it was identified through endocrinologic screening. All patients with alterations in the pituitary-adrenal axis presented with abnormalities in baseline blood tests and with signs and symptoms suggestive of adrenal insufficiency. Patients with endocrinologic deficits received diagnoses reported in Tables and Figures, Supplemental Digital Content 1–29, http://links.lww.com/INF/F912 and standard treatments.^9–15^

The brain MRI performed during the follow-up showed alterations in the hypothalamic-pituitary region in the last 5 cases described, which also corresponded to the cases defined as more severe. Patients with previous meningitis had a higher risk of developing endocrine dysfunction compared to other types of infection (8 cases versus 3 cases in encephalitis and 1 in the abscess group, *P =* 0.005).

Subsequently, we analyzed the results of hematologic and hormonal tests conducted during the endocrinological follow-up of these patients, comparing the results with those of the control group. The previously infected patients had different levels of centrally produced hormones such as TSH (*P* < 0.001) and ACTH (*P* < 0.001); cortisol was also significantly different between the 2 cohorts (*P =* 0.019).

IGF-1 was significantly lower in patients with previous CNS infection, both in absolute terms and in relation to standard deviations by sex and age (both *P* < 0.001).

We also conducted an analysis by subgroups, comparing patients based on the different types of CNS infection; again, we found statistically significant differences in IGF-1 median values and their IQR, TSH, levels of antithyroid peroxidase antibodies, ACTH, cortisol, LH, serum potassium, CD3 and CD4. Specifically, patients with prior meningitis had lower levels of IGF-1 compared to other categories. Summary of our results and relative *P* values are specified in Tables and Figures, Supplemental Digital Content 1–29, http://links.lww.com/INF/F912.

We subsequently analyzed the differences between various groups, classifying patients based on the etiology of the pathogen causing the CNS infection (bacteria, virus, mycobacteria and other agents such as fungi). In accordance with our previous results, we confirmed a statistical difference in IGF-1 and their standard deviations, TSH, ACTH and LH, highlighting also a difference in aldosterone values; *P* values are reported in Table 3.

**TABLE 3.** Differences in Hormonal Values Based on the Etiology of CNS Infection

	Total	Controls	Bacteria	Virus	Mycobact	Other	
	N = 80	N = 27	N = 27	N = 23	N = 2	N = 1	*P* value
IGF-1	205.3 (144.3)	323.6 (149.3)	158.3 (113.0)	120.5 (70.7)	164.0 (84.9)	309.0 (.)	**<0.001**
IGF-1 SD	−0.7 (1.1)	0.1 (1.0)	−1.3 (1.0)	−0.9 (1.0)	−0.8 (1.3)	−0.2 (.)	**<0.001**
GH	0.4 (0.2–1.4)	0.4 (0.2–0.5)	0.2 (0.2–1.4)	1.1 (0.2–2.4)	1.3 (0.2–2.4)	0.1 (0.1–0.1)	0.35
TSH	2.6 (1.4)	3.4 (1.4)	1.9 (1.4)	2.7 (1.2)	1.6 (0.3)	2.2 (.)	**0.002**
fT3	4.0 (0.8)	4.2 (0.5)	3.7 (1.1)	4.0 (0.8)	4.2 (0.5)	4.8 (.)	0.17
fT4	11.9 (2.1)	12.2 (1.7)	11.9 (2.8)	11.4 (1.6)	10.3 (0.3)	15.1 (.)	0.24
Ab Anti-TPO	36.9 (35.7)	29.8 (4.5)	40.1 (47.1)	42.8 (42.5)	28.0 (0.0)	28.0 (.)	0.73
Ab AntiTG	2.0 (3.3)	1.7 (1.5)	1.3 (0.0)	3.2 (5.9)	1.3 (0.0)	1.3 (.)	0.37
ACTH	22.8 (11.6)	31.8 (7.5)	16.7 (10.3)	20.4 (11.4)	14.0 (4.2)	15.0 (.)	**<0.001**
Cortisol	93.2 (49.0)	108.8 (34.5)	82.7 (56.2)	85.0 (48.1)	63.0 (35.4)	202.0 (.)	0.031
Prolactine	7.2 (4.5)	6.9 (2.8)	6.6 (3.5)	8.7 (6.8)	4.2 (2.9)	6.2 (.)	0.41
FSH	2.7 (2.4)	2.4 (2.0)	3.6 (3.0)	2.0 (1.6)	3.1 (3.1)	2.0 (.)	0.16
LH	1.4 (2.0)	1.2 (1.7)	2.2 (2.5)	0.5 (1.1)	2.3 (3.2)	5.7 (.)	**0.006**
Sodium	137.7 (2.9)	137.0 (1.6)	138.6 (4.3)	137.6 (1.7)	137.0 (2.8)	139.0 (.)	0.37
Potassium	4.1 (0.4)	4.0 (0.3)	4.0 (0.4)	4.3 (0.5)	3.8 (0.6)	3.9 (.)	**0.036**
Plasma osmolarity	291.8 (3.3)	292.0 (3.5)	292.1 (3.4)	290.9 (2.7)	295.0 (7.1)	290.0 (.)	0.4
Glucose	82.0 (7.7)	82.5 (8.1)	82.5 (6.0)	81.0 (9.2)	85.5 (0.7)	73.0 (.)	0.66
Insuline	8.1 (6.3)	7.3 (4.2)	8.9 (7.0)	8.7 (7.9)	5.1 (3.7)	5.4 (.)	0.8
HOMA-IR	1.7 (1.5)	1.6 (0.9)	1.8 (1.4)	1.8 (2.0)	1.1 (0.8)	1.0 (.)	0.87
Renine	42.2 (31.3)	44.9 (23.6)	42.9 (44.4)	40.2 (22.0)	28.8 (3.1)	26.1 (.)	0.92
Aldosterone	124.9 (99.8)	104.8 (53.9)	100.4 (52.0)	186.7 (151.6)	70.0 (53.7)	20.0 (.)	**0.007**

Bold value indicates statistically significant values.

**FIGURE 1.**
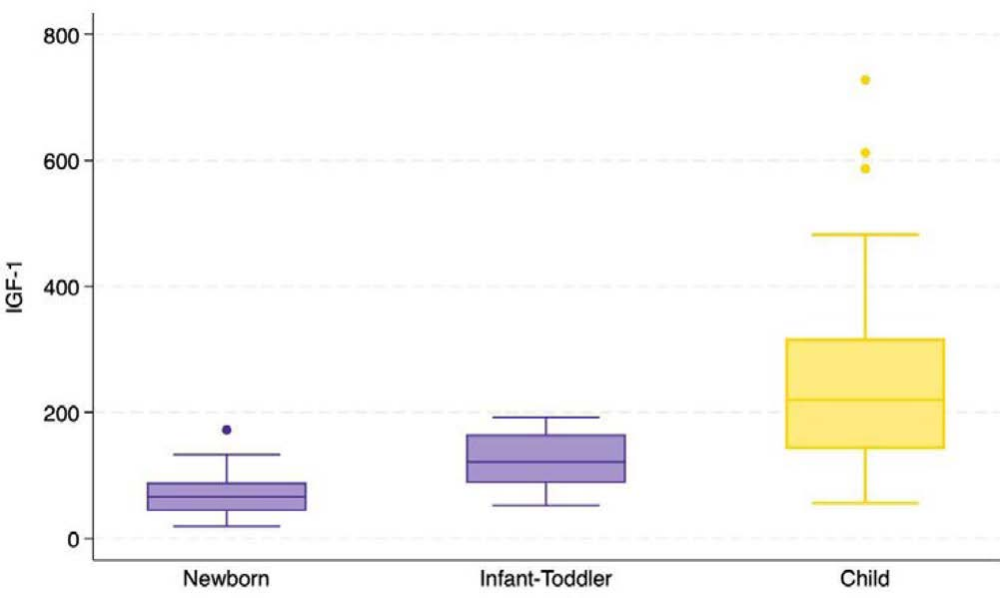
IGF-1 values according to different ages.

Finally, we analyzed the various hormone levels based on the age of onset of the CNS infection, to evaluate whether neonates with early-onset CNS infections exhibit different hormone levels compared to patients who contracted infections at a later age (Fig. 1). We found a statistical significant difference in the absolute value of IGF-1, although not confirmed by the analysis of standard deviations. Additionally, the group of patients with neonatal infection had significantly lower levels of ACTH compared to the group of patients with infection during infancy and in older children, while they had higher levels of Vitamin D. More information is provided in Tables and Figures, Supplemental Digital Content 1–29, http://links.lww.com/INF/F912.

### Regression Models

We also applied multivariable linear regression models to evaluate the association between the biomarkers and auxologic parameters with relevant covariates.

IGF-1 was associated with height Z-scores in univariate analysis but never in multivariable analysis; body mass index Z-scores were not associated with IGF-1 in both uni- and multivariable analysis. They were associated with puberty in a negative relationship. It was associated with puberty and age at the time of infection. It was also associated with TSH (close to significance). More information are reported in Tables and Figures, Supplemental Digital Content 1–29, http://links.lww.com/INF/F912.

**FIGURE 2.**
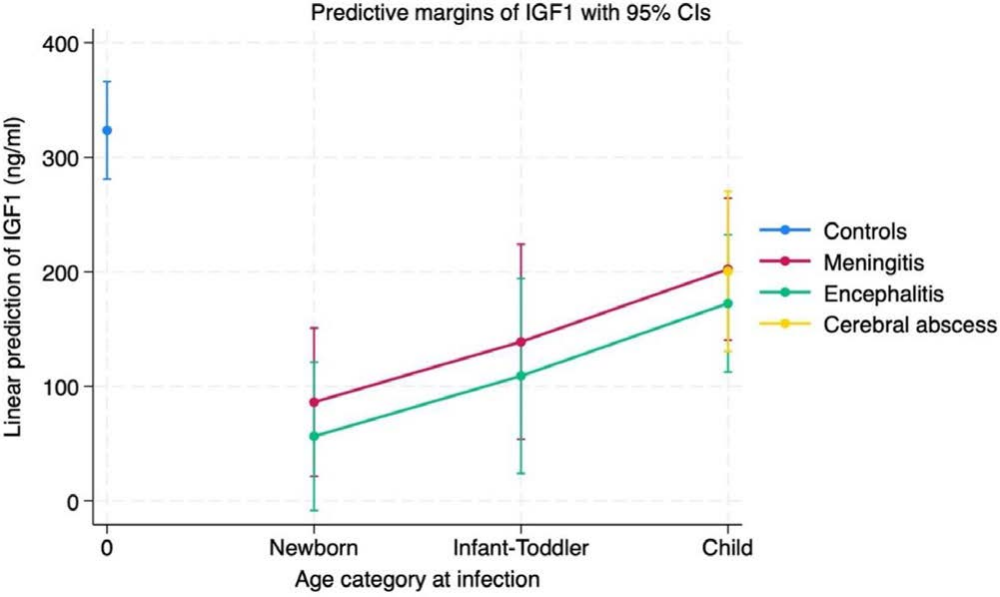
Predictive values of IGF-1 according to age category at infection and type of infection.

Hormone levels were also studied with multivariable linear regression including in the model the age at the time of infection, temporal distance from the infection, kind of infection and type of microorganism and having had a neurological deficit because of infection (Fig. 2).

From our multivariable linear regression analysis (Table 4), the reduction in IGF-1 was greater the earlier the CNS infection occurred, regardless of the type of infection contracted. The presence of a concurrent neurological deficit following the infection was found to be associated with lower levels of IGF-1. IGF-1 also does not correlate with TSH, in uni- and multivariable analysis.

**TABLE 4.** Multivariable Analysis with IGF-1 Values

IGF-1	Coefficient	Std. err.	t	P>t	[95% conf. interval]
Age category at infection					
Newborn	−**239.27**	55.73	−4.29	<0.001	−350.31 to −128.22
Infant-Toddler	−**186.59**	62.25	−3	0.004	−310.64 to −62.55
Child	−**123.23**	41.08	−3	0.004	−205.08 to −41.38
Infection type					
Meningitis	1.87	46.87	0.04	0.968	−91.53 to 95.27
Encephalitis	−27.97	46.23	−0.6	0.547	−120.07 to 64.14
Cerebral abscess	0	(omitted)			
_cons	323.64	21.36	15.15	0	281.08–366.19

Bold value indicates statistically significant values.

Vitamin D levels presented correlations with temporal distance from infection (Coeff: −1.78; 95% CI: −3.18 to −0.378; *P* = 0.014) but not with presenting neurologic deficits.

Regarding LH levels, a significant correlation was found with the age of infection in patients with a history of meningitis and encephalitis, but not in those with a history of brain abscess. The presence of a neurologic deficit was not associated with LH levels.

## DISCUSSION

In this study, we found that children of different clusters of CNS infections diagnosed at different ages are characterized by several neuropsychiatric and endocrinologic sequelae. To our knowledge, this is the most detailed follow-up analysis of children with different types of CNS infections.

Regarding follow-up, periodic neuropsychiatric evaluations are essential to assess the onset of motor, sensory, or neurosensory outcomes, which vary in frequency based on clinical characteristics, the affected brain site, and the underlying etiological agent. Although meningitis tends to cause intracranial organic alterations more frequently than encephalitis, some studies report an incidence of neurosensory deficits of up to 50% in patients with herpesvirus infection.^16^

Endocrinologic follow-up is less standardized, as there are no common guidelines and recommendations.^17^ Our study is one of the first to investigate the incidence of endocrinological alterations in a cohort of children with a history of CNS infection. Our findings indicate that children with a history of CNS infection, regardless of the type of infection, exhibit lower height compared to healthy controls (assessed both as an absolute value and as a standard deviation for age and sex), along with significantly lower IGF-1 levels, associated with height standard deviations in univariate analysis. Furthermore, patients with bacterial meningitis are at a higher risk than those with other CNS infections for developing neuropsychiatric and endocrinologic complications both in the short and long term, and they also exhibit the lowest plasma IGF-1 levels among the groups studied. Severe previous bacterial infections are factors that could negatively affect the growth rate of children^18^; children who survive infections such as bacterial meningitis often exhibit nutritional deficits and metabolic problems that can contribute to reduced growth compared to their healthy peers.^19^ Nutritional impairment negatively affects IGF-1 levels,^20^ leading to a reduction in growth hormone action and a potential decrease in growth velocity that can also be attributed to a general compromise of the children’s overall condition due to prolonged hospital stays and physical limitations resulting from postinfectious neurological alterations.^21,22^ Additionally, organic conditions such as the onset of hydrocephalus and postinflammatory changes affecting the hypothalamic-pituitary region also play a significant role.^4^

We also observed differences in the levels of other pituitary hormones, including ACTH, TSH, and LH, which were lower compared to healthy controls. Despite our results, differences in these values were not considered clinically significant. This is primarily because these hormones are typically assessed using absolute values, unlike IGF-1, which is evaluated with Z-scores adjusted for age and sex. For many of these hormonal markers, there are currently no universally accepted reference tables to determine pathological values that consider patient-specific factors such as age and sex; consequently, clinicians often rely on arbitrary cutoffs to define abnormal levels, which may not accurately reflect the normal range for all individuals,^9^ potentially leading to misinterpretation or inappropriate management in certain cases.

A recent study^5^ analyzed the endocrinological hormonal profile, both basal and stimulated, of 19 adults (13 males and 6 females) with a past CNS infection, between 22 and 65 years of age (mean 38.7 ± 11.7 years) and with known etiology (4 neuroborreliosis, 2 tick-borne encephalitis, 1 herpes simplex, 1 varicella, 1 enterovirus, 10 of unknown origin but classified as meningitis); all patients were evaluated at least 6 months after the acute episode, presenting with neurosensory sequelae ranging from mild to moderate, in the absence of signs or symptoms suggestive of endocrinopathy. The authors analyzed basal levels of GH, IGF-I, ACTH, cortisol, gonadotropins, testosterone (in males), estradiol, and progesterone (in females), as well as TSH, fT3, fT4, and prolactin, while subsequently undergoing an insulin tolerance test (ITT) with standard dosage. Hormonal evaluation yielded results like those of our analysis, as 4 patients (21%) showed isolated corticotropin insufficiency (peak cortisol <181.25 μg/L during ITT). Two patients (11%, males) showed borderline gonadotropin insufficiency (basal testosterone between 2.4 and 3.0 μg/L), while in no case was a deficiency of ADH, GH or TSH evident. Even though an ITT was not performed on our patients, we observed lower ACTH levels in patients with a history of CNS infection, with the lowest levels found in those with a history of meningitis. However, it is known that ACTH is a hormone that can be influenced by various factors, including stress.^23^ Therefore, isolated alterations in corticotropin levels, in the absence of changes in cortisol levels or symptoms suggestive of adrenal insufficiency, have limited clinical relevance. In our institute, when there is a suspected covert corticosteroid deficiency with normal baseline cortisol levels, a second-level stimulation test is typically performed in a short time frame. This involves administering synthetic ACTH and measuring cortisol levels before and after the injection at 30- and 60-minute intervals. Conversely, in the case of pathologic values in the absence of suggestive symptoms, baseline hormone levels are repeated, and only afterward are second-level tests performed.

Another difference observed in our analysis was the more frequent occurrence of gonadotropin activation, in the form of precocious puberty, rather than gonadotropin deficiency in our pediatric patients.

Overall, we diagnosed 3 cases of central precocious puberty, and in all 3 of our patients, this condition was associated with other endocrinological disorders. The patients presented with elevated basal LH levels (typically <0.1 IU/L in prepubertal subjects) and progression of secondary sexual characteristics, including the onset of thelarche. Among the pituitary disorders we analyzed, central precocious puberty is the only condition associated with increased hormone secretion (of GnRH) rather than a deficiency. One possible hypothesis is that inflammatory stimuli during childhood (when the axis is suppressed) may lead to activation, whereas in adulthood, with an active GnRH axis, it may cause negative feedback. Both inhibition and stimulation of the gonadal axis, however, fall within the spectrum of possible inflammatory alterations following CNS infections.^7^

Another analysis like ours was conducted by Levy-Shraga et al.^24^ examining the basal hormonal function of 14 patients with prior meningoencephalitis (3 cases of confirmed bacterial meningitis) contracted during pediatric age between 2 weeks and 15.8 years. They found that in 3 cases, IGF-1 levels were below −2 standard deviations for age and sex; of these, only 1 patient had a growth velocity below normal limits, and none of the 3 patients were subsequently diagnosed with growth hormone deficiency. While these results highlight that a prior inflammatory stimulus may sometimes cause a deficit in pituitary hormonal secretion, it is worth considering that IGF-1 is the effector of growth hormone,^11^ it is not produced at the pituitary level, and it is also influenced by the clinical and nutritional status of the patients.^25^ Therefore, it is possible that in the 3 cases described by the authors, the reduction in IGF-1 below normal limits may be attributable to other causes.

In our statistical analysis, we highlighted how IGF-1 levels in children with previous CNS infection are lower than controls; GH deficiency emerged also as the most frequent endocrinological disorder (5 cases, of which 3 were isolated and 2 combined with another deficit); these observations are consistent with the sensitivity level of the adenohypophyseal cells, as somatotroph cells are the most sensitive to pathogenic stimuli and are compromised early in case of inflammatory lesions or space-occupying processes within the intracranial space, leading to hypothalamic-pituitary damage.^26,27^

Our analysis also revealed that patients who contracted the infection in the neonatal period had lower IGF-1 levels compared to children who acquired the CNS infection at a later age. However, the analysis of standard deviations for sex and age did not confirm this association, rendering this result clinically insignificant. Although meningitis is more frequent and severe when contracted in the neonatal period,^28,29^ our subgroup analysis did not show that patients with neonatal CNS infections have a higher rate of endocrine complications.

In the multivariable linear regression, IGF-1 levels were found to be significantly lower in patients who contracted the infection early, with no differences observed between the various types of infections. Additionally, IGF-1 levels were significantly lower in patients with associated neurologic deficits.

Regardless of the absence or presence of confirmed endocrinologic deficits, we have highlighted also that levels of certain centrally produced hormones such as ACTH were lower in the cohort of patients with prior CNS infection compared to the control group; despite the reduction, hormone levels were still within the normal range for age; therefore, these patients were not subjected to second-level tests to search for underlying endocrinological pathology. Indeed, hypopituitarism following a harmful stimulus can be partial and not clinically manifest, not requiring replacement pharmacological therapies in the absence of clinical symptoms.^19,30,31^

Our study has some limitations. It is a single-center study conducted in an Italian hospital, and our results may not align with international findings, particularly given the varying prevalence of bacterial and viral pathogens. Although the endocrinological follow-up is prospectively organized, the data for this study have been collected retrospectively. Not all patients underwent second-level testing for endocrinopathies, and it is possible that performing dynamic tests, even in the absence of symptoms, could lead to the diagnosis of additional asymptomatic endocrinopathies in our patients, such as subacute-onset forms of adrenal insufficiency. It was not possible, in the end, to perform an analysis evaluating the incidence of endocrinological alterations based on the specific etiological agent causing CNS infection, as the considered subgroups turned out to be too small to provide adequate statistical significance. However, we observed that, in the meningitis group, 2 cases were associated with a previous infection by Streptococcus pneumoniae, 2 with Escherichia coli, 2 with *Klebsiella pneumoniae*, while respectively 1 with *Streptococcus intermedius* and 1 with *Citrobacter koseri.*

Nevertheless, this study remains the most detailed endocrinological follow-up of children who survived different types of CNS infections diagnosed in different age groups.

In conclusion, our study provided information about several endocrinological imbalances in children who survived CNS infections. Among the various types of infections, meningitis carries the highest risk of subsequent endocrinopathies, particularly short stature. Encephalitis and cerebral abscesses are rarely associated with subsequent endocrinopathies and generally cause endocrine disorders only in the presence of anatomical alterations of the hypothalamic-pituitary axis. These data highlight the importance of including long-term outcomes in any interventional study of children with CNS infections.
